# Supplementary material for: Titanium Implant Surface Effects on Adherent Macrophage Phenotype: A Systematic Review
Source: Materials (Basel). 2022 Oct 19;15(20):7314. doi: 10.3390/ma15207314 (PMC9609829; doi:10.3390/ma15207314)
Supplement: Supplementary file 1 [file materials-15-07314-s001.zip › materials-1913252-supplementary.pdf]

materials

Supplementary data

Titanium Implant Surface Effects on Adherent Macrophage Phenotype: A Systematic Review

Manju Pitchai, Deepak Ipe, Santosh Tadakamadla and Stephen Hamlet *

School of Medicine and Dentistry, Griffith University, Gold Coast Campus, Southport, QLD 4222, Australia;
* Correspondence: s.hamlet@griffith.edu.au (S.H.); Tel.: +61 (0)7-5678-0483

Table S1: ToxRTOOL assessment tool [16]. A binary score (1 or 0) was determined for each of eighteen reliability criteria.

Ref	Author, year	Test substance	Purity of test	Source of test	Nature of test	Test system	Source of test	Information of test	Administration	Concentration	Time points	Negative controls	Positive controls	Replicates of test	End points described	End points investigation	Statistical of test	Substance specific	Result reliability	Total
**[13]**	Pan, 2017	1	1	1	1	1	1	1	1	1	1	0	1	1	1	1	1	1	1	17
**[18]**	Milleret, 2011	1	1	1	1	1	1	1	1	1	1	0	1	0	1	1	1	1	1	16
**[19]**	Hamlet ,2012	1	1	1	1	1	1	1	1	1	1	1	1	1	1	1	1	1	1	18
**[20**	Barth, 2013	1	0	1	1	1	1	1	1	1	1	0	1	0	1	1	1	1	1	15
**[21]**	Alfarsi, 2014	1	1	1	1	1	1	1	1	1	1	0	1	1	1	1	1	1	1	17
**[22]**	Nagasawa, 2015	1	1	1	1	1	1	1	1	1	1	1	1	1	1	1	1	1	1	18
**[23]**	Hotchkiss, 2016	1	1	1	1	1	1	1	1	1	1	1	1	1	1	1	1	1	1	18
**[24]**	Eger, 2017	1	1	1	1	1	1	1	1	1	1	1	1	1	1	1	1	1	1	18
**[25]**	Hotchkiss, 2017	1	1	1	1	1	1	1	1	1	1	1	1	1	1	1	1	1	1	18
**[26]**	Kinoush, 2017	1	1	1	1	1	1	1	1	1	1	0	1	1	1	1	1	1	1	17
**[27]**	Choi, 2018	1	1	1	1	1	1	1	1	1	1	0	0	1	1	1	1	1	1	16
**[28]**	Hotchkiss, 2018	1	1	1	1	1	1	1	1	1	1	1	1	1	1	1	1	1	1	18
**[29]**	Yang, 2018	1	1	1	1	1	1	1	1	1	1	0	1	0	1	1	1	1	1	16
**[30]**	Becker, 2019	1	1	1	1	1	1	1	1	1	1	1	1	1	1	1	1	1	1	18
**[31]**	Hamlet, 2019	1	1	1	1	1	1	1	1	1	1	0	1	1	1	1	1	1	1	17
**[32]**	Hotchkiss, 2019	1	1	1	1	1	1	1	1	1	1	0	1	1	1	1	1	1	1	17
**[33]**	Zhu, 2019	1	1	1	1	1	1	1	1	1	1	0	1	0	1	1	1	1	1	16
**[34]**	Ma, 2014	1	1	1	1	1	1	1	1	1	1	0	1	1	1	1	1	1	1	17
**[35]**	Wang, 2017	1	1	1	1	1	1	1	1	1	1	0	1	1	1	1	1	1	1	17
**[36]**	Wang, 2018	1	1	1	1	1	1	1	1	1	1	1	1	0	1	1	1	1	1	17
**[37]**	Ma, 2018	1	1	1	1	1	1	1	1	1	1	0	1	1	1	1	1	1	1	17
**[38]**	Chen, 2020	1	1	1	1	1	1	1	1	1	1	0	1	1	1	1	1	1	1	17
**[39]**	Li, 2020	1	1	1	1	1	1	1	1	1	1	0	1	1	1	1	1	1	1	17
**[40]**	Bai, 2018	1	1	1	1	1	1	1	1	1	1	0	1	1	1	1	1	1	1	17
**[41]**	Bai, 2018	1	1	1	1	1	1	1	1	1	1	0	1	1	1	1	1	1	1	17
**[42]**	Zhang, 2018	1	1	1	1	1	1	1	1	1	1	1	1	1	1	1	1	1	1	18
**[43]**	Takebe, 2007	1	1	1	1	1	1	1	1	1	1	1	1	1	1	1	1	1	1	18
**[44]**	Scislowska-Czarnecka, 2012	1	1	1	1	1	1	1	1	1	1	0	1	0	1	1	1	1	1	16
**[45]**	Nayak, 2013	1	1	1	1	1	1	1	1	1	1	1	1	0	1	1	1	1	1	17
**[46]**	Wu, 2014	1	1	1	1	1	1	1	1	1	1	0	1	0	1	1	1	1	1	16
**[47]**	Wu, 2015	1	1	1	1	1	1	1	1	1	1	1	1	0	1	1	1	1	1	17
**[48]**	Huang, 2016	1	1	1	1	1	1	1	1	1	1	1	1	1	1	1	1	1	1	18
**[49]**	Ryden, 2017	1	1	1	1	1	1	1	1	1	1	1	1	1	1	1	1	1	1	18

materials

**[50]**	Araujo-Gomes, 2019	1	1	1	1	1	1	1	1	1	1	1	1	1	1	1	1	1	1	18
**[51]**	Chen, 2020	1	1	1	1	1	1	1	1	1	1	1	1	0	1	1	1	1	1	17
**[52]**	Morra, 2015	1	1	1	1	1	1	1	1	1	1	1	1	1	1	1	1	1	1	18
**[53]**	Zhang, 2018	1	1	1	1	1	1	1	1	1	1	0	1	1	1	1	1	1	1	17
**[54]**	He, 2019	1	1	1	1	1	1	1	1	1	1	1	1	1	1	1	1	1	1	18
**[55]**	Zhang, 2019	1	1	1	1	1	1	1	1	1	1	0	1	1	1	1	1	1	1	17

Table S2: Synthesis without Meta-analysis (SWiM) reporting items [59].

**Reporting item**	**Item description**	**Page in manuscript**
Methods		
**1** Grouping studies for synthesis	1a) Provide a description of, and rationale for, the groups used in the synthesis (e.g., groupings of populations, interventions, outcomes, study design)	Study design – pg. 3, 4
	1b) Detail and provide rationale for any changes made subsequent to the protocol in the groups used in the synthesis	Data extraction – pg. 4, 5
**2** Describe the standardised metric and transformation methods used	Describe the standardised metric for each outcome. Explain why the metric(s) was chosen, and describe any methods used to transform the intervention effects, as reported in the study, to the standardised metric, citing any methodological guidance consulted	Data extraction – pg. 4, 5
**3** Describe the synthesis methods	Describe and justify the methods used to synthesise the effects for each outcome when it was not possible to undertake a meta-analysis of effect estimates	Data extraction – pg. 4,5
**4** Criteria used to prioritise results for summary and synthesis	Where applicable, provide the criteria used, with supporting justification, to select the particular studies, or a particular study, for the main synthesis or to draw conclusions from the synthesis (e.g., based on study design, risk of bias assessments, directness in relation to the review question)	Data extraction – pg. 4, 5
**5** Investigation of heterogeneity in reported effects	State the method(s) used to examine heterogeneity in reported effects when it was not possible to undertake a meta-analysis of effect estimates and its extensions to investigate heterogeneity	Semiquantitative analysis and summary statistics – pg. 5, 7, 10, 13
**6** Certainty of evidence	Describe the methods used to assess certainty of the synthesis findings	Table 3 – pg. 7 & 8

**7** Data presentation methods	Describe the graphical and tabular methods used to present the effects (e.g., tables, forest plots, harvest plots). Specify key study characteristics (e.g., study design, risk of bias) used to order the studies, in the text and any tables or graphs, clearly referencing the studies included	Summary study data provided over 6 tables. Risk of bias – supplementary table 1.
Results		
**8** Reporting results	For each comparison and outcome, provide a description of the synthesised findings, and the certainty of the findings. Describe the result in language that is consistent with the question the synthesis addresses, and indicate which studies contribute to the synthesis	Surface topography – pg. 5<br>Cell Morphology – pg. 7<br>Cellular Response – pg. 10
Discussion		
**9** Limitations of the synthesis	Report the limitations of the synthesis methods used and/or the groupings used in the synthesis, and how these affect the conclusions that can be drawn in relation to the original review question	Discussion - pg. 16, 17

Glossary of abbreviations used:

ASA	Aspirin
BG	Bioglass
CS	Calcium silicate
cpTi	commercially pure titanium
GG07-I	0.7% genipin hydrogel + IL4
GG07	0.7% genipin hydrogel - IL4
HA	Hydroxyapatite
HA/cpTi	Hydroxyapatite coating on commercially pure titanium
MAO	Micro-arc oxidation,
M/cpTi	Machine polished cpTi,
Micro	Micro-scale roughness,
modSLA	Hydrophilic sand-blasted and acid-etched
Nano	Nano-scale roughness
NT5	Titanium nanotube anodized at 5V

NT20	Titanium nanotube anodized at 20V
NTPS	Nanotube plasma-sprayed
PT	Polished titanium
RXD	Roxolid
SLActive	Hydrophilic SLA
SBA)	Hyper hydrophilic sand-blasted and acid-etched
S/cpTi	Specular polished cpTi
SLA)	Sand-blasted and acid-etched
SMS	Strontium-Magnesium-Silicone coating
SrSLA	Strontium SLA
TCPS	Tissue culture plastic surface
TNT	Titanium nanotube
Ti-NW	Nanowire-modified titanium surface
TiNW-Zn	Zinc-containing nanowires,
Ti-SF/LL-37	Titanium-peptide LL-37-loaded silk fibroin nanoparticles
Ti-SS),	Titanium sericin
Ti-SS-RGD	Titanium sericin arginine-glycine-aspartate
TPS	Plasma-sprayed titanium coating
